# C-LTMRs evoke wet dog shakes via the spinoparabrachial pathway

**DOI:** 10.1126/science.adq8834

**Published:** 2024-11-07

**Authors:** Dawei Zhang, Josef Turecek, Seungwon Choi, Michelle Delisle, Caroline Leal Pamplona, Shan Meltzer, David D. Ginty

**Affiliations:** 1 Department of Neurobiology, Howard Hughes Medical Institute, Harvard Medical School, 220 Longwood Avenue, Boston, MA 02115, USA

## Abstract

Many hairy mammals perform rapid oscillations of their body, called wet dog shakes, to remove water and irritants from their back hairy skin. The somatosensory mechanisms underlying this behavior are unclear. We report that Piezo2-dependent mechanosensation mediates wet dog shakes evoked by water or oil droplets applied to back hairy skin of mice. Unmyelinated low-threshold mechanoreceptors (C-LTMRs) were activated by oil droplets and their optogenetic activation elicited wet dog shakes. Ablation of C-LTMRs attenuated this behavior. Moreover, C-LTMRs synaptically couple to spinoparabrachial neurons, and optogenetically inhibiting spinoparabrachial neuron synapses and excitatory neurons in the parabrachial nucleus impaired both oil droplet- and C-LTMR-evoked wet dog shakes. Thus, a C-LTMR–spinoparabrachial pathway promotes wet dog shakes for removal of water and mechanical irritants from back hairy skin.

## Main text

Wet dog shakes are an evolutionarily conserved behavior observed widely across hairy mammalian species. This behavior consists of rapid oscillations of the head and upper trunk, typically following exposure of back hairy skin of animals to water and other irritating or potentially damaging stimuli (1–3). Despite the prevalence of wet dog shakes and range of stimuli that can elicit them (4–11), the neurobiological mechanisms underlying this highly conserved, stereotypical behavior have remained elusive.

More than twelve physiologically and morphologically distinct primary somatosensory neuron subtypes innervate the hairy skin of mammals (12). These cutaneous sensory neurons collectively detect and encode a range of environmental stimuli, with individual subtypes exhibiting unique stimulus-response profiles (13–15). Even simple mechanical stimuli, such as static (step) indentation or brushing of the skin, can activate several mechanosensory neuron subtypes that exhibit distinct response properties. For example, hairy skin Aβ rapidly adapting (RA) low-threshold mechanoreceptors (LTMRs) fire at the onset and offset of static indentation and respond strongly to rapid brushing or vibrating the skin, and are thus dynamic touch detectors (11, 13, 16). In contrast, unmyelinated C-fiber LTMRs (C-LTMRs) exhibit sustained responses to static indentation and strong responses to slowly moving stimuli (13, 16–18). Therefore, C-LTMRs are poised to report the persistence of gentle, sustained mechanical stimuli acting on hairy skin. The contributions of these and other hair follicle-associated LTMRs and other cutaneous sensory neuron subtypes to the wet dog shake response is unknown.

How somatosensory signals encoding stimuli acting on mammalian hairy skin are transformed in the central nervous system (CNS) into motor commands that coordinate the wet dog shake is also an open question. All somatosensory neuron subtypes terminate within specific laminae of the spinal cord dorsal horn where they synapse onto different combinations of spinal cord neurons. Spinal projection neurons then convey somatosensory information to other CNS regions to generate motor commands, autonomic responses, and sensory perception (19, 20). The identity of dorsal horn neurons and their corresponding output pathways that initiate wet dog shakes are unknown. Here, we have used mouse genetic, physiological, and behavioral approaches to address the somatosensory neurobiological basis of wet dog shakes.

## Results

### Oil droplets applied to the neck trigger wet dog shakes via a Piezo2-dependent mechanism

**Fig. 1.**
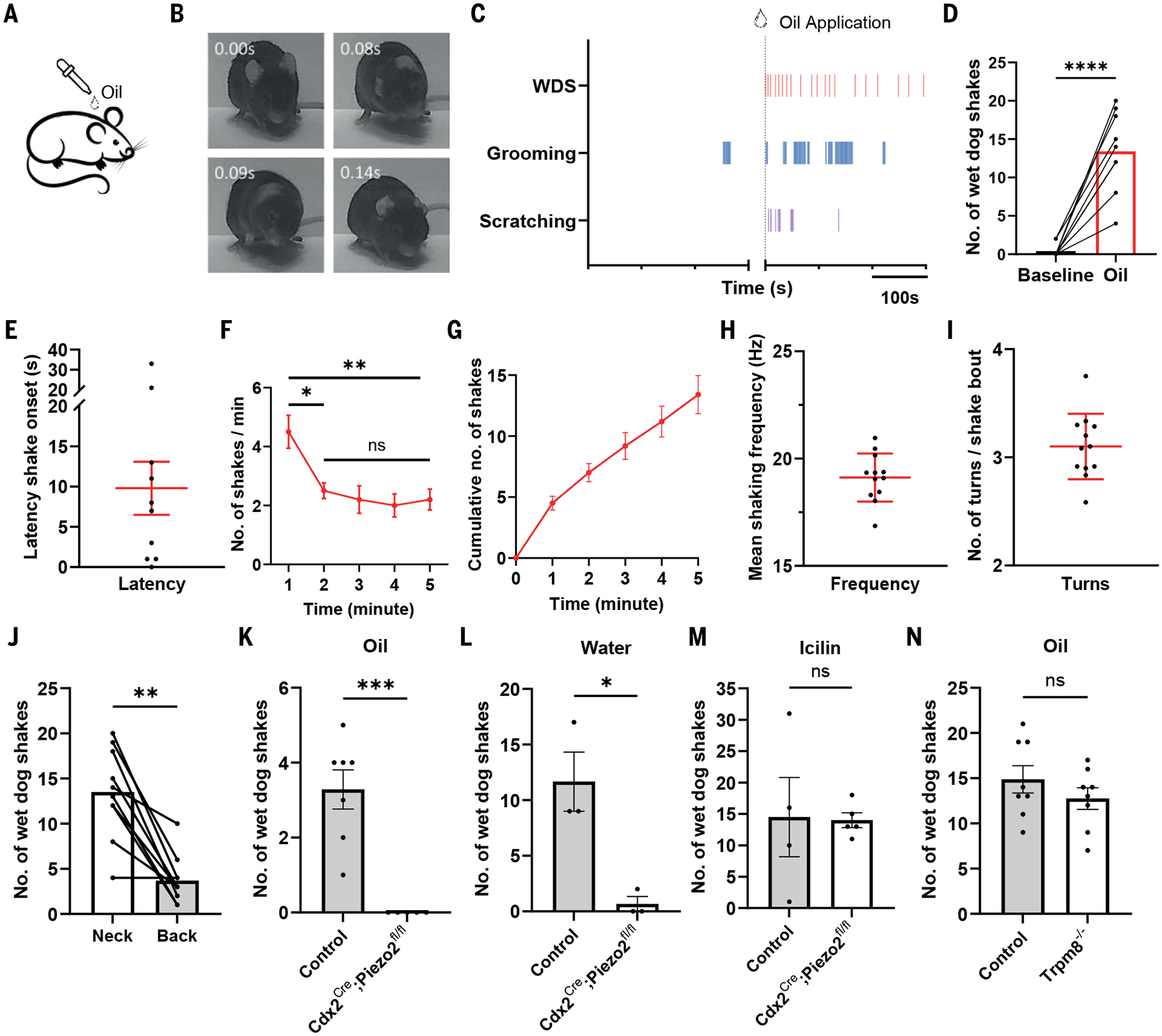
Oil droplets trigger wet dog shakes via Piezo2-mediated mechanosensation. (**A**) Schematic of oil droplet applied to a mouse’s neck. (**B**) Representative video frames of oil droplet-evoked wet dog shakes (WDS). Time relative to shake onset. (**C**) Representative raster plots of WDS, grooming, and scratching episodes before and after an oil droplet application. (**D**) Bar graphs of total number of WDS across animals (C57BL/6, N = 10) [two-sided paired t-test]. (**E-G**) WDS onset latencies (E), average counts per minute (F) [One-way ANOVA], and cumulative numbers (G) following oil droplet treatment across animals shown in (D). (**H** and **I**) Average shaking frequencies (H) and average number of oscillatory turns per shaking bout (I) across animals (N = 12). Error bar: SD. (**J**) Number of WDS when oil droplets were applied to back versus neck (N = 10) [two-sided paired t-test]. (**K**-**M**) Number of oil droplet- (K), water- (L), and icilin- (M) induced WDS in control (oil: N = 7; water: N =3; icilin: N = 4) and Piezo2 conditional knockout animals (oil: N = 5; water: N=3; icilin: N = 5, same animal as in (K)) [two-sided unpaired t-tests]. Control animals lacked either the *Cre* allele or one copy of the floxed *Piezo2* allele. (**N**) Number of oil droplet-induced WDS in control (C57BL/6, N = 8) and *Trpm8* knockout animals (N = 8) [two-sided unpaired t-test]. Shown are the means ± SEM, except in (H) and (I). ns p > 0.05, * p < 0.05, ** p < 0.01, *** p < 0.001, **** p < 0.0001. Black dots represent individual animals.

Wet dog shakes are observed widely across mammalian species in response to different stimuli (4–11). To begin to elucidate somatosensory mechanisms underlying this stereotypical behavior, we first tested a range of somatosensory stimuli for their ability to evoke wet dog shakes in mice. We observed that swimming in a water bath, spraying water onto the animal’s fur (1, 3) (fig. S1A), applying a sunflower seed oil droplet, gentle air puff, or thin von Frey filaments to the back of the neck, and chloroquine injection into neck hairy skin all triggered wet dog shakes (Fig. 1A, fig. S1B–D). Similar to the water bath and water spray stimuli, sunflower seed oil droplets applied to the back of the neck evoked wet dog shake bouts with comparable body motion kinetics in nearly all animals tested (Fig. 1B, D, fig. S1E, F, movie S1). Because of its reliability, ease of application, and precise spatiotemporal control, we used the oil droplet stimulus to investigate the contribution of different somatosensory neuron subtypes and central circuitry underpinnings of this stereotypical mammalian behavior (Fig. 1A).

Oil droplets applied to the back of the neck induced many bouts of wet dog shakes over a five-minute recording period, and this was often accompanied by scratching and grooming (Fig. 1C, D, fig. S1G, K). On average, a single oil droplet evoked wet dog shakes with a latency of ~10 seconds, although this varied across animals and trials (Fig. 1E). Animals responded most robustly during the first minute following oil droplet application and subsequently performed fewer shakes (Fig. 1F, G). In contrast, grooming behaviors persisted throughout the recording period, whereas the timing of scratching varied across animals (fig. S1H, I, L, M). Individual wet dog shake episodes were highly stereotyped across animals, with a mean frequency of oscillatory movements of 19.12 Hz (SD = 1.12) and an average of 3.10 (SD = 0.31) full back and forth turns per shaking bout (Fig. 1H, I). Animals were more responsive to oil droplets applied to the back of the neck, displaying more wet dog shakes and scratching bouts compared to oil droplets applied to the lower back. Wet dog shakes were not observed when oil droplets were applied to the thigh (Fig. 1J, fig. S1J, N, O).

Stimulus-evoked wet dog shakes may reflect responses to different sensory modalities (4–11, 21). Indeed, wet dog shakes evoked by liquids including water or oil droplets may reflect responses to mechanical or thermal stimuli or both (3). To test whether mechanosensation is the primary driver of water- and oil droplet-induced wet dog shakes (3), the mechanosensitive ion channel Piezo2 was deleted in all dorsal root ganglion (DRG) neurons below the upper cervical region of the body using *Cdx2*^*Cre*^*;Piezo2*^*fl/fl*^ mice (11, 22, 23) and responses to water bath or oil droplet application to the back of the neck were examined. We observed an absence of oil droplet-evoked wet dog shakes (Fig. 1K) and a nearly complete loss of water-evoked wet dog shakes in the *Piezo2* mutants (Fig. 1L). The lack of responses in *Piezo2* mutant mice is due to insensitivity to mechanical stimuli rather than an inability to perform wet dog shakes because intraperitoneal injection of icilin, which is an agonist of the cold/cool-sensitive ion channel TRPM8 (24–26), evoked wet dog shakes to a comparable extent in *Piezo2* mutants and control mice (Fig. 1M, fig. S1P, Q, movie S2). Control experiments showed that animals lacking TRPM8 (*Trpm8*^*−/−*^) (27) and wildtype controls exhibited comparable bouts of wet dog shakes evoked by oil droplets (Fig. 1N), whereas icilin-induced wet dog shakes were lost in *Trpm8*^*−/−*^ animals (28) (fig. S1R). Thus, water- and oil droplet-evoked wet dog shakes require Piezo2-dependent mechanosensation.

### Low threshold mechanoreceptors are preferentially activated by oil droplets applied to hairy skin

**Fig. 2.**
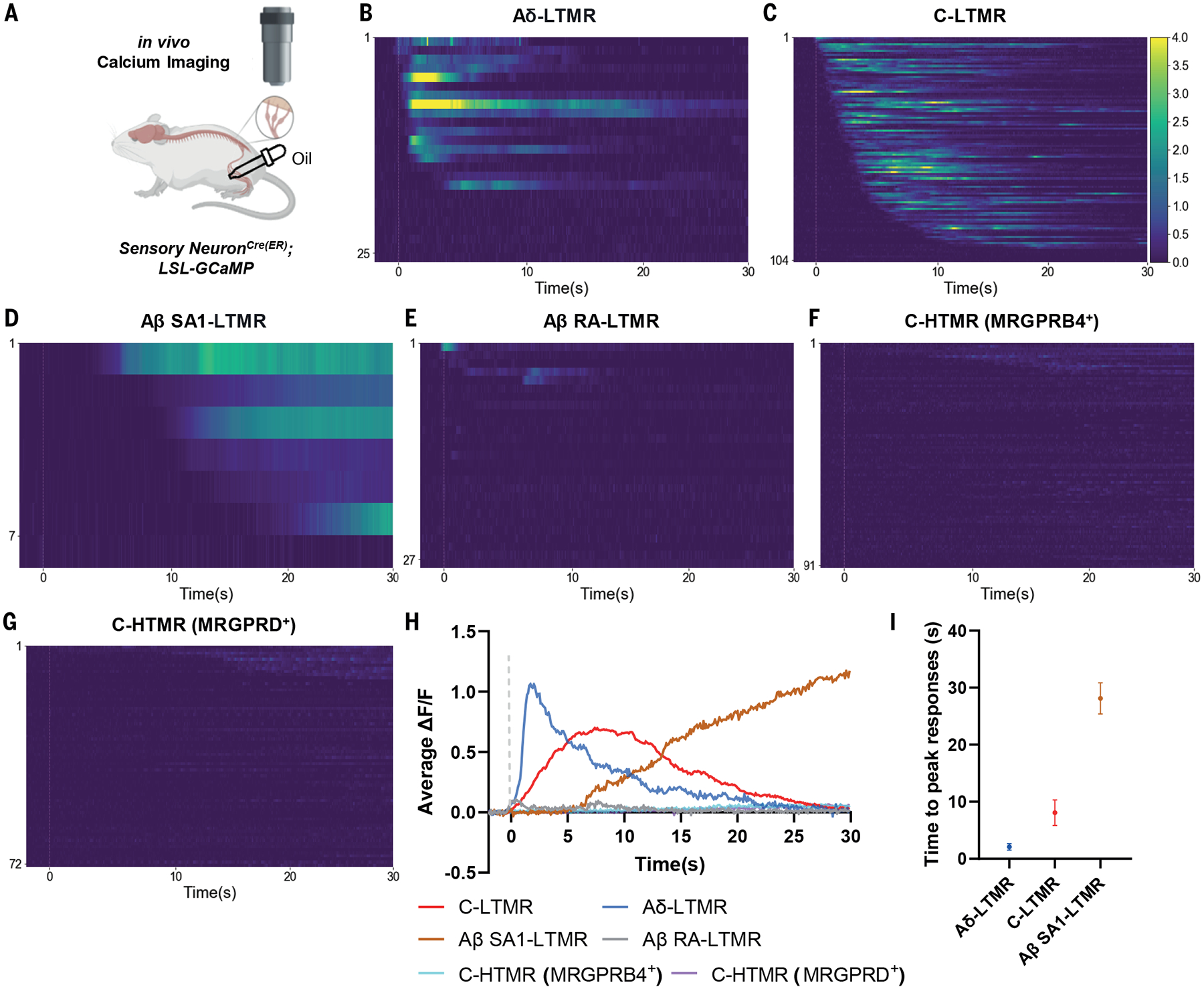
Select LTMR subtypes are preferentially activated by oil droplets applied to hairy skin. (**A**) Schematic of *in vivo* DRG calcium imaging experiments with oil droplet stimuli. (**B-G**) Heatmaps of calcium responses in (B) Aδ-LTMRs (*TrkB*^*CreER*^ *(Ntrk2*^*CreER*^*); Ai96*, N = 3, n = 25), (C) C-LTMRs (*Th*^*CreER*^*; Ai148*, N = 3, n = 104), (D) Aβ SA1-LTMRs (*TrkC*^*CreER*^ *(Ntrk3*^*CreER*^*); Ai148*, N = 2, n = 7), (E) Aβ RA-LTMRs (*Ret*^*CreER*^*; Ai148*, N = 3, n = 27), (F) C-HTMRs (MRGPRB4^+^), (*Mrgprb4*^*CreER*^*; Ai148*, N = 3, n = 91), and (G) C-HTMRs (MRGPRD^+^) (*Mrgprd*^*CreER*^*; Ai148*, N = 3, n = 72) in response to oil droplet. Rows of the heatmap represent responses of individual DRG neurons over time. (**H**) Average calcium responses of Aδ-LTMRs, C-LTMRs, Aβ SA1-LTMRs, Aβ RA-LTMRs, C-HTMRs (MRGPRB4^+^), and C-HTMRs (MRGPRD^+^) in response to oil droplets applied to the skin. (**I**) Time to reach peak calcium responses in Aδ-LTMRs, C-LTMRs, and Aβ SA1-LTMRs after oil droplet application. Shown are the means ± SEM. Dashed lines indicate the oil droplet application time. N: number of animals. n: number of cells.

We next sought to identify the primary mechanosensory neurons that mediate mechanically evoked wet dog shakes. *In vivo* DRG calcium imaging experiments were conducted to assess responses across the principal mechanosensory neuron subtypes following application of oil droplets to hairy skin (Fig. 2A). Due to the physical challenges of accessing cervical DRGs that innervate hairy skin of the neck and the unavoidable damage to the skin and sensory nerve endings during cervical DRG surgical exposure, we conducted experiments on lumbar L4 DRGs and applied oil droplets to hairy skin of the thigh. We examined the responsiveness of six distinct DRG mechanosensory neuron types, including four low threshold mechanoreceptors (LTMRs): Aβ RA-LTMRs, Aβ slowly-adapting type 1 (SA1)-LTMRs, Aδ-LTMRs, and C-LTMRs; and the two most mechanically sensitive C-fiber high threshold mechanoreceptors (HTMRs): C-HTMRs expressing MRGPRB4 (MRGPRB4^+^) and C-HTMRs expressing MRGPRD (MRGPRD^+^) (14). In agreement with previous findings (11, 13, 14, 29–32), all four LTMR types and both C-HTMR types responded to brushing or pinching of the skin (fig. S2). Application of oil droplets to hairy skin evoked robust responses in most C-LTMRs, Aδ-LTMRs, and Aβ SA1-LTMRs (Fig. 2B–D), whereas few Aβ RA-LTMRs, C-HTMRs (MRGPRB4^+^), and C-HTMRs (MRGPRD^+^) responded to the stimulus (Fig. 2E–G). Averaging population responses across the sensory neuron types showed that C-LTMRs, Aδ-LTMRs, and Aβ SA1-LTMRs exhibited responses with distinct temporal dynamics (Fig. 2H, I), whereas Aβ RA-LTMRs, C-HTMRs (MRGPRB4^+^), and C-HTMRs (MRGPRD^+^) exhibited virtually no responses to the oil droplet stimulus (Fig. 2H). Thus, C-LTMRs, Aδ-LTMRs, and Aβ SA1-LTMRs are candidate drivers of mechanical stimulus-evoked wet dog shakes.

### Optogenetic stimulation of C-LTMRs induces wet dog shakes

**Fig. 3.**
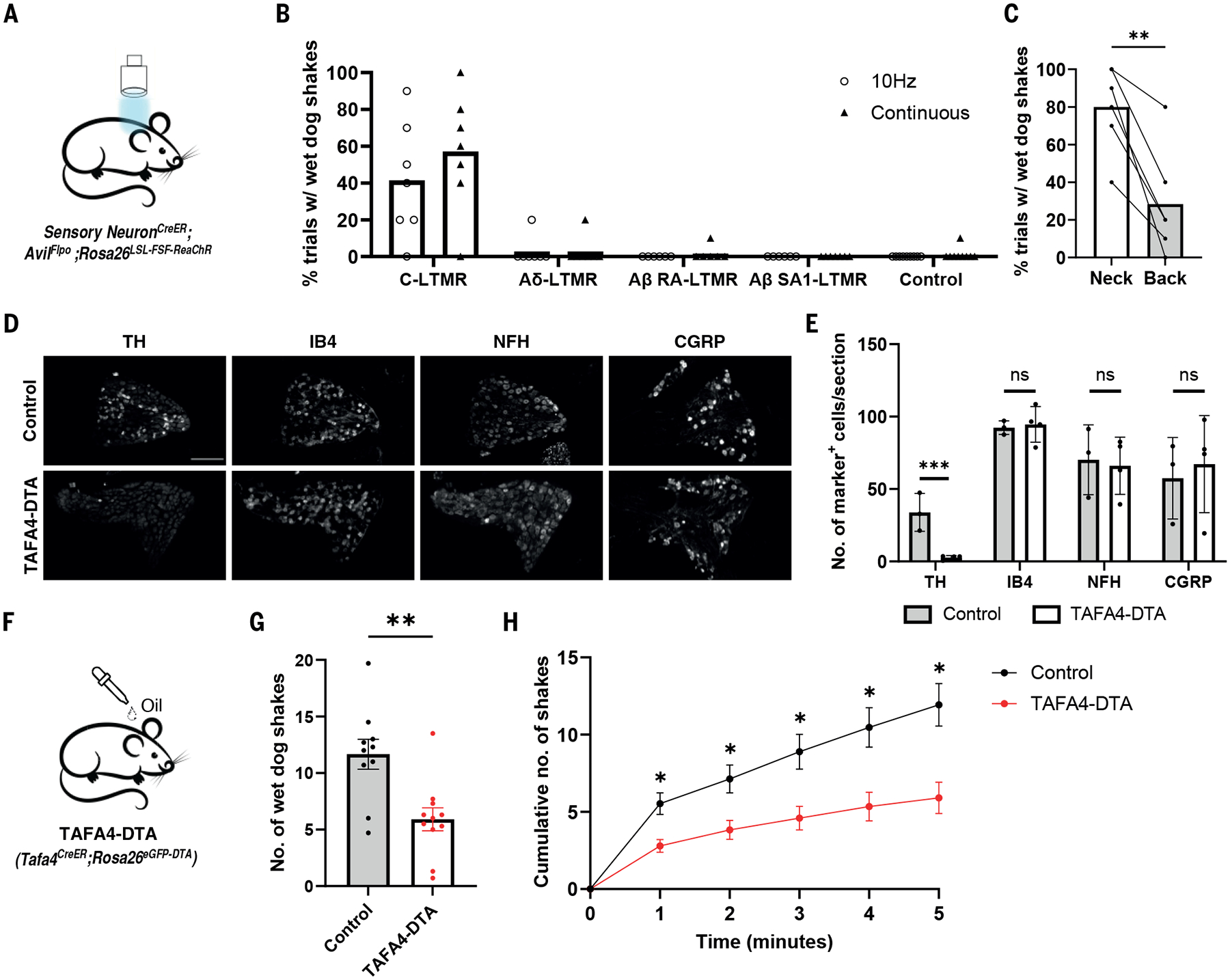
Optogenetic stimulation of C-LTMRs evokes wet dog shakes whereas ablation of C-LTMRs attenuates oil droplet-induced wet dog shakes. (**A**) Schematic of photostimulation of sensory neurons innervating the skin. (**B**) Percent trials with WDS induced by photostimulation of LTMR types. Number of animals tested: *Th*^*CreER*^*; Avil*^*Flpo*^*; Rosa26*^*LSL-FSF-ReaChR*^, N = 7. *TrkB*^*CreER*^*; Avil*^*Flpo*^*; Rosa26*^*LSL-FSF-ReaChR*^, N = 6. *Ret*^*CreER*^*; Avil*^*Flpo*^*; Rosa26*^*LSL-FSF-ReaChR+*^, N = 6. *TrkC*^*CreER*^*; Avil*^*Flpo*^*; Rosa26*^*LSL-FSF-ReaChR*^, N = 7. Controls are littermates without the CreER or Flpo allele (N = 9). (**C**) Percent trials with WDS induced by 2s continuous light exposure on the neck versus back in *Th*^*CreER*^*; Avil*^*Flpo*^*; Rosa26*^*LSL -FSF-ReaChR*^ animals (N = 6, including 2 ReaChR allele homozygotes) [two-sided paired t test]. (**D** and **E**) Representative images (D) [scale bars = 200 μm] and quantification (E) of cervical DRG neurons stained with cell type markers in control (*Rosa26*^*eGFP-DTA*^, N = 3) and TAFA4-DTA (N = 4) animals [two-sided unpaired t-test]. Dots represent individual animals. (**F**) Schematic of oil droplet application to the neck of TAFA4-DTA animals. (**G**) Number of WDS induced by oil droplet application to littermate control (without *Tafa4*^*CreER*^ allele, N = 10) and TAFA4-DTA animals (N = 11) [two-sided unpaired t-test]. Dots represent averaged responses from at least 2 repeated trials of an animal. (**H**) Cumulative plot of the number of WDS over time [Two-way ANOVA]. Shown are the means ± SEM. ns p > 0.05, * p < 0.05, **p < 0.01, ***p < 0.001.

To determine whether stimulation of one or more LTMR subtypes is sufficient to evoke wet dog shakes, we expressed the light-activated cation channel ReaChR (33, 34) in the four LTMR subtypes and activated them by illuminating skin of the neck or back while monitoring mice for wet dog shakes (Fig. 3A, fig. S3A). Optogenetic stimulation of neurons labeled using *Th*^*T2a-CreER*^, which are C-LTMRs and sympathetic neurons, consistently elicited wet dog shakes (Fig. 3B, movie S3). These responses were mediated by C-LTMRs and not sympathetic neurons because optogenetic stimulation of sympathetic neurons alone failed to evoke wet dog shakes (fig. S3B–C). Optogenetic stimulation of Aδ-LTMRs, Aβ SA1-LTMRs, and Aβ RA-LTMRs failed to elicit wet dog shakes (Fig. 3B, fig. S3D), despite our findings that these optical stimuli can evoke robust neural responses in the spinal cord (35, 36).

We further explored C-LTMR-evoked wet dog shake responses and found that, as with oil droplet-evoked responses, optogenetic stimulation of C-LTMRs terminating in dorsal neck hairy skin triggered more pronounced wet dog shakes compared to stimulation of C-LTMRs terminating in hairy skin of the lower back, and photostimulation of the thigh did not evoke any response (Fig. 3C, fig. S3E). This difference in behavioral responses was not due to variations in C-LTMR density, terminal morphology, or genetic labeling specificity or efficiency across the skin regions (fig. S4). Moreover, characteristic features of wet dog shakes, including shaking frequency and the number of body turns per bout, were comparable between those evoked by oil droplet application and C-LTMR photostimulation (fig. S3F, G). Additionally, as with oil droplet evoked responses, the onset latency of wet dog shakes following C-LTMR optostimulation varied across trials (fig. S3H). Wet dog shakes were most frequently initiated towards the end of the 10 Hz light stimulation sessions (fig. S3I) and in some cases (~19%) commenced after cessation of light stimulation (fig. 3J).

### Ablation of C-LTMR neurons reduces wet dog shakes

Because of the robust behavioral responses observed following C-LTMR optogenetic stimulation, we next asked whether C-LTMRs are necessary for wet dog shakes induced by the oil droplet stimulus. To this end, we generated a new mouse line *Tafa4*^*CreER*^ (fig. S5A), which labels C-LTMRs but not sympathetic neurons (37) (fig. S5B–E). As expected, optogenetic stimulation of C-LTMRs labeled using *Tafa4*^*CreER*^ induced wet dog shakes (fig. S5F). We crossed this line to *Rosa26*^*eGFP-DTA*^ mice to generate *Tafa4*^*CreER*^; *Rosa26*^*eGFP-DTA*^ mice (hereafter referred to as TAFA4-DTA mice) to ablate C-LTMRs and not other primary sensory neurons. Adult tamoxifen treatment resulted in ~92% reduction of C-LTMRs in TAFA4-DTA mice, as determined by TH antibody staining of DRGs. Moreover, C-LTMRs were selectively lost, as DRG neuronal populations marked by IB4^+^, CGRP^+^, and NFH^+^ did not differ from control mice lacking the *Tafa4*^*CreER*^ allele (Fig. 3D, E).

We observed ~50% reduction in oil droplet-evoked wet dog shakes in TAFA4-DTA mice compared to controls, a finding consistent across trials (Fig. 3F–H, fig. S7A). To mitigate potential confounds associated with TAFA4 expression elsewhere in the body (fig. S5G–K), we also ablated C-LTMRs along with other C-fiber neurons and some Aδ-fiber HTMRs in the DRG using *Scn10a*^*Cre*^ *;Rosa26*^*eGFP-DTA*^ animals (hereafter referred to as SNS-DTA mice) (38) (fig. S6A, B). SNS-DTA mice mirrored the TAFA4-DTA animals, exhibiting ~48% reduction in oil-induced wet-dog shakes (fig. S6C, D), suggesting that the reduction of responses seen in TAFA4-DTA animals was due to the loss of C-LTMRs.

**Fig. 4.**
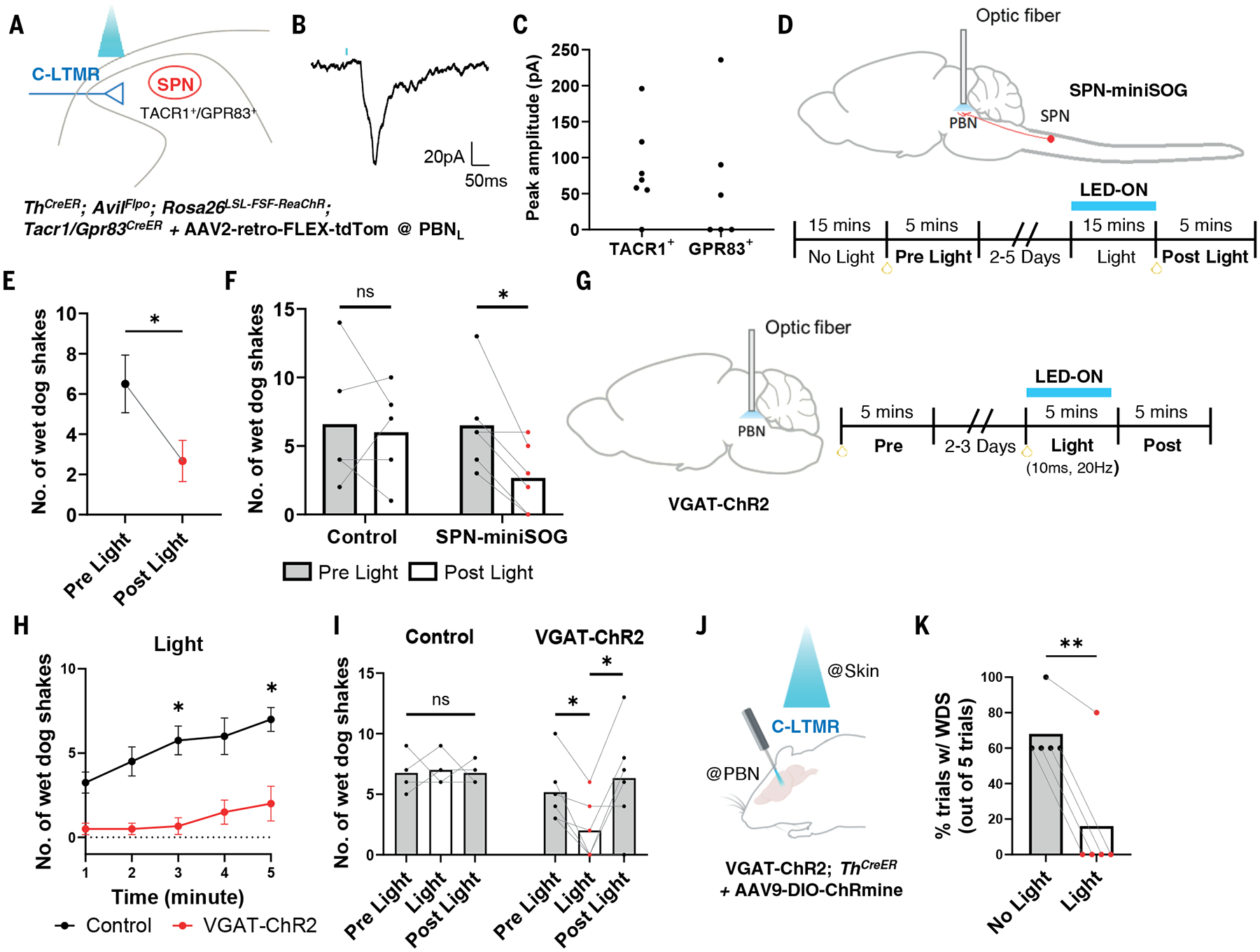
The spinoparabrachial pathway mediates oil droplet- and C-LTMR-evoked wet dog shakes. (**A**) Schematic of genetic labeling strategy and spinal cord slice whole cell recordings from SPNs during photostimulation of TH^+^ primary afferent terminals. (**B**) Representative trace of a light-activated EPSC from an SPN. Blue line indicates light onset. (**C**) Peak amplitudes of light induced EPSCs in TACR1^+^ and GPR83^+^ SPNs (TACR1^+^, n = 8; GPR83^+^, n = 7). (**D**) Schematic of photoinhibition of synaptic release from SPN terminals and the behavioral experimental design. (**E**) Average number of oil-induced WDS before and after light stimulation of SPN-miniSOG animals (N = 6). (**F**) Individual responses in littermate control (lack either *Lbx1*^*Flpo*^ or *Rosa26*^*LSL-FSF-SYP1-miniSOG-mcherry*^ allele, N = 5) and SPN-miniSOG animals. (**G**) Schematic of photoinhibition of PBN neurons using VGAT-ChR2 animals and the experimental design. (**H**) Cumulative plot of average number of WDS in control (C57Bl/6, N = 4) and VGAT-ChR2 animals (N = 6) during PBN light stimulation (3mW) [two-way ANOVA]. (**I**) Number of oil-induced WDS before, during, and after light stimulation in control and VGAT-ChR2 animals from (H) [two-sided paired t-test]. **(J)** Schematic of genetic labeling strategy and experimental design for simultaneous light activation of C-LTMR endings in the skin and VGAT^+^ inhibitory inputs to the PBN. (**K**) Percent trials with light- (on the skin) induced WDS with and without VGAT-ChR2 photoactivation in the PBN (N = 5) [two-sided paired t-test]. N: number of animals. n: number of cells. Dots represent individual animals except in (C) and (E). Shown are the means ± SEM. Bars represent means. * p < 0.05, **p < 0.01.

To further assess somatosensory and motor behaviors in TAFA4-DTA mice, we performed a series of additional behavioral measurements. Our experiments revealed normal locomotor activity in these animals using the open field and balance beam assays (fig. S7B, C). Moreover, icilin-induced wet-dog shakes were normal in TAFA4-DTA mice (fig. S7D), indicating that the wet dog shake motor pattern generation function remains intact in these mice. Also, TAFA4-DTA mice did not exhibit differences in grooming behaviors in response to oil droplets compared to their littermate controls (fig. S7E). TAFA4-DTA mice exhibited ~58% reduction in oil-induced scratching compared to controls (fig. S7F), but these mice did not exhibit alterations in scratching in response to histamine or chloroquine (fig. S7G–H). In addition, TAFA4-DTA mice exhibited normal cold thermosensation, which was assessed using a temperature preference assay (fig. S7I), and these mutants also showed normal withdrawal responses to von Frey filaments applied to their hind paw (fig. S7J, K). Collectively, these findings demonstrate that C-LTMRs contribute to oil droplet-evoked wet dog shakes but are not required for a range of other somatosensory behaviors. Residual wet dog shake responses observed in TAFA4-DTA mice (Fig. 4F–H, fig. S7A) may reflect sufficiency of the small number (~8%) of remaining C-LTMRs in these mutants or the contribution of one or a combination of other LTMRs in evoking the behavior.

### The spinoparabrachial pathway mediates wet dog shakes.

The finding that C-LTMRs contribute to stimulus-evoked wet dog shakes afforded an opportunity to ask how these mechanosensory neurons engage central circuits to mediate somatosensory behaviors. C-LTMRs are known to terminate in lamina IIiv of the superficial spinal cord dorsal horn (16, 17). We hypothesized that C-LTMR signals are conveyed from the superficial dorsal horn to the lateral parabrachial nucleus (PBN_L_) via spinoparabrachial (SPB) neurons to mediate wet dog shakes. To investigate this possibility, we first asked whether optogenetic activation of the central terminals of C-LTMRs could evoke postsynaptic responses SPB neurons. We performed whole-cell patch-clamp electrophysiological slice recordings of TACR1^+^ and GRP83^+^ SPB neurons (SPNs), which together account for ~90% of SPB neurons of the superficial dorsal horn (39) (Fig. 4A). We found that C-LTMRs are indeed synaptically coupled to SPNs, since optogenetic activation of C-LTMR terminals evoked excitatory postsynaptic currents (EPSCs) in both SPN populations (Fig. 4B, C).

To ask whether SPNs mediate mechanically evoked wet dog shake behaviors, we used the light-activated protein miniSOG (40) to inhibit SPN synaptic transmission in the PBN_L_. Fusion of miniSOG to synaptophysin (SYP1) enables light-triggered oxidation of synaptic proteins necessary for triggering neurotransmitter release and synaptic transmission (40). We generated a dual recombinase-dependent mouse line, *Rosa26*^*FSF-LSL-SYP1-miniSOG*^ that enables Cre and FlpO recombinase-dependent expression of SYP1-miniSOG at synaptic terminals (fig. S8A) and thus inhibition of synaptic transmission using optical stimuli applied to axon terminals. Consistent with previous findings (40), light application to spinal cord excitatory neuron axon terminals expressing SYP1-miniSOG suppressed excitatory drive by ~75% (fig. S8B–D). Therefore, we expressed SYP1-miniSOG in SPNs using *Tacr1*^*CreER*^*; Gpr83*^*CreER*^*; Lbx1*^*Flpo*^*; Rosa26*^*FSF-LSL-SYP1-miniSOG*^ mice (hereafter referred to as SPN-miniSOG mice) (39) and implanted optical fibers into the PBN to enable light-evoked inhibition of SPN synaptic transmission (Fig. 4D). Upon light stimulation of the PBN, SPN-miniSOG mice exhibited ~58% reduction in oil droplet-evoked wet dog shakes (Fig. 4E, F). In contrast, wet dog shakes were unperturbed following light stimulation of the PBN in control animals. SPN-miniSOG mice exposed to light did not exhibit a statistically significant reduction in oil droplet-induced scratching behaviors or grooming behaviors (fig. S9A–D). In addition, light stimulation of SPN-miniSOG animals did not alter one other aspect of back hairy skin tactile reactivity, as motor responses measured using a tactile pre-pulse inhibition (tPPI) assay (41, 42) were unaffected (fig. S9E, F).

To complement the findings using SPN-miniSOG mice and determine whether C-LTMR-mediated wet dog shakes require the SPB pathway, we silenced the PBN directly, through optogenetic stimulation of GABAergic neurons of the PBN using VGAT-ChR2 animals and assessed the consequences of this manipulation on oil droplet- and C-LTMR-evoked wet dog shakes. Consistent with the SPN-miniSOG manipulations, broad inhibition of the PBN through optogenetic stimulation of PBN local and projecting GABAergic neurons inhibited wet dog shakes evoked by both oil droplets (Fig. 4G–I) and photostimulation of C-LTMR terminals in the skin (Fig. 4J, K, fig. S9G). Collectively, these findings demonstrate that a C-LTMR–spinoparabrachial pathway contributes to mechanically evoked wet dog shakes.

## Discussion

Wet dog shakes are an evolutionarily conserved behavior observed across mammals that serves to remove water and other irritants from back and neck hairy skin, a skin region that is largely unreachable by self-grooming or -licking. Here, we report that a C-LTMR–spinoparabrachial pathway contributes to mechanically-evoked wet dog shakes.

The contributions of C-LTMRs to perception and behavior has been a matter of considerable interest. A prevailing view is that human C-mechanoreceptors mediate affective, pleasurable touch, an idea stemming from human microneurography recordings and psychophysical studies (43–45). C-LTMRs are the most sensitive C-fiber mechanoreceptors in mice and are often considered the murine equivalent of human C-mechanoreceptors implicated in affective touch (14, 45, 46). In mice, however, other C-fiber mechanoreceptor populations that do not innervate hair follicles and have higher force thresholds may also contribute to affective touch (30, 47). Our findings implicate C-LTMRs as mediators of wet dog shakes, although C-LTMR loss-of-function experiments suggest that they may not be the only DRG sensory neuron mediating this behavior. Related to this, in the seminal work identifying C-LTMRs nearly 80 years ago (48), Zotterman suggested that C-LTMRs may underlie the sensation of ‘tickle.’ In support of Zotterman’s notion, we observed that C-LTMR activation evokes wet dog shakes, and animals with fewer C-LTMRs exhibit a reduction in both oil droplet evoked wet dog shakes and scratching, suggesting that C-LTMR activation may indeed evoke a tickle or mechanical irritation sensation. It is also noteworthy that C-LTMRs form lanceolate axonal endings exclusively around the small vellus hairs of the body (16), which comprise the undercoat, an insulating layer of hair that may serve to protect against environmental irritants and threats. Thus, we propose that C-LTMRs detect the lightest forces acting on hairy skin, including water, movements of insects and parasites, and other stimuli that deflect vellus hairs, triggering motor behaviors that have evolved to remove irritants or potential threats.

Our findings show that SPB neurons play a key role in conveying C-LTMR signals to the brain to initiate mechanically evoked wet dog shakes. It is noteworthy that wet dog shakes can be elicited by stimuli that span different sensory modalities, including cooling agents and pruritogens (fig. S1D, R), and photoactivation of at least two other C-fiber sensory neurons with distinct response properties, the C-HTMR (MRGPRB4^+^) and C-Cold (TRPM8^+^) neurons, can evoke wet dog shakes (fig. S10). Moreover, the SPB pathway is implicated in behavioral responses to both thermal stimuli and pruritogens (39, 49–52). Therefore, we suspect that the PBN mediates wet dog shakes evoked by stimuli across a range of sensory modalities. Future research will aim to reveal the PBN neuronal ensembles that drive wet dog shakes, and how PBN signals are relayed to downstream brain regions and central motor pattern generation centers to initiate this conserved, stereotypical behavior.

## Supplementary Material

Supplementary Movie S1

Supplementary Movie S2

Supplementary Movie S3

Supplementary Material

## Data Availability

All data are available in the manuscript or the supplementary material. Reagents are available from the corresponding author upon reasonable request. Customized codes are available to download from GitHub (https://github.com/DZneuro/WDS).
